# Limitations in ROP Programs in 32 Neonatal Intensive Care Units in Five States in Mexico

**DOI:** 10.1155/2015/712624

**Published:** 2015-06-17

**Authors:** L. Consuelo Zepeda-Romero, Clare Gilbert

**Affiliations:** ^1^Clinic of Retinopathy of Prematurity, Hospital Civil de Guadalajara, Centro Universitario de Ciencias de la Salud, Universidad de Guadalajara, 44280 Guadalajara, JAL, Mexico; ^2^Department of Clinical Research, Faculty of Infectious Tropical Diseases, London School of Hygiene & Tropical Diseases, London WC1E 7HT, UK

## Abstract

Retinopathy of prematurity (ROP) is the main cause of avoidable blindness in children in Mexico despite National ROP Guidelines and examination of preterm infants being a legal requirement.* Objective*. To assess coverage of ROP programs and their compliance with national guidelines.* Study Design*. Thirty-two neonatal intensive care units (NICUs) in five of the largest states were visited. Staff were interviewed to collect information on their ROP programs which were defined as (1) compliant, if National Guidelines for screening and treatment were followed, (2) noncompliant, if other approaches were used, or (3) no program.* Results*. Only 10 (31.2%) had fully compliant programs and 11 (34.4%) had no program. In the remaining 11 (34.4%) different screening criteria were used (7 units): screening was undertaken by an ophthalmologist in unsalaried time (4), was not undertaken in the NICU (2), and was undertaken by a neonatologist (1) and/or Avastin was used as first-line treatment (7). Poorer states had poorer programs.* Conclusions*. Despite legislation mandating eye examination of preterm births, many ROP programs in the largest cities in Mexico require improvement or need to be established. Prevention of blindness due to ROP needs to be prioritized in Mexico to control the epidemic of ROP blindness.

## 1. Introduction

Recent estimates, which used statistical modelling of data on preterm birth rates, access to neonatal care and survival rates, rates of retinopathy of prematurity (ROP) requiring treatment, the proportion of these infants treated, and the outcome of treatment, suggest that 32,000 preterm infants (uncertainty range 24,800–44,500) became blind or visually impaired globally in the year 2010 from ROP [1]. These estimates are higher than those of a decade ago, which were based on childhood blindness prevalence estimates and the proportion of blindness due to ROP [2]. The more recent estimate suggests that Asia has the highest incidence of blindness due to ROP, followed by Latin America where 3,500 (uncertainty range 2,600–5,200) infants a year are affected [1]. Analysing the data per million live births shows that Latin American countries have a 2.4 times higher incidence of ROP blindness/severe visual impairment than highly industrialized countries. The study also suggests that approximately 50% of infants developing sight threatening ROP globally were not treated in 2010.

Retinopathy of prematurity is the commonest cause of blindness in children in many middle income countries [3], including Mexico. A recent study in Guadalajara, the capital of Jalisco State, Mexico, in which 153 blind or severely visually impaired children in two schools for the blind were examined, showed ROP to be the commonest cause of blindness (34.7%) [4]. The proportion of children less than 10 years of age who were blind from ROP was higher than those aged 10–15 years (42.3% versus 18.8%), which suggests that ROP is becoming a more important cause over time. Almost half of these children (46%) had not been treated for ROP.

The control of visual impairment from ROP requires high quality neonatal care from the first few minutes after birth, coupled with programs for the detection and treatment of infants who develop the constellation of clinical signs which indicate a high rate of progression to blinding ROP [5, 6]. Program guidelines need to take account of the population of infants at risk, which vary by location [3]. Many middle income countries have national guidelines (e.g., Brazil [7], Argentina [8], Chile [9], Ecuador, India [10], El Salvador [11], China [12], Peru [13], and Russia) and have prioritized control in National Prevention of Blindness plans. In recognition that blindness from ROP is a major avoidable cause of blindness in children in Latin America, the Pan American Health Organization (PAHO) has prioritized ROP for control in the region [14–17]. Mexico has National ROP Guidelines [18], and legislation has been passed making examination of preterm infants mandatory [19]. The Mexican Guidelines recommend that infants with birth weights (BW) ≤1,750 g or gestational age (GA) ≤34 weeks are screened, that is, taking account of the risk in bigger, more mature infants in middle income settings. The guidelines were updated in 2010 but did not include indications for and possible adverse events associated with intravitreal injection of anti-VEGF agents [18], nor is it recommended as a primary treatment.

The study in Guadalajara outlined above also investigated the presence and type of ROP programs in the 17 neonatal intensive care units (NICUs) in the city, five being in the private sector [4]. Nine NICUs, including all five in the private sector, had ROP programs which complied with national guidelines. The remaining eight units had programs which did not comply in one or more respects; that is, infants were screened in an eye clinic (3 units); parents took their infant to another NICU for examination after discharge (3 units), or the neonatologist screened infants at risk (1 unit). At the time of the study 390 preterm infants could receive care in these NICUs but only 60% of the 318 cots outside the private sector had a compliant ROP program.

Mexico has a population of 112 million, with over 2 million births in 2010, over 51,000 (2.5%) being preterm (≤34 weeks of GA). The majority of the preterm infants were born in the public sector (87%), and in all states most NICUs are in the state capitals [20]. Mexico has a complex health system with several different health providers. The majority of the population now has access to health insurance schemes, and in 2007 the Popular Insurance scheme covered costs of neonatal intensive care and treatment of ROP [21]. Despite improvement in neonatal care and access to insurance, infants with Stage 5 continue to present to the ROP Clinic in the Hospital Civil de Guadalajara, many coming from outside Jalisco State [22]. The purpose of this study was to extend the earlier study, to assess the presence and quality of programs for detecting and treating ROP in neonatal units in five states in Mexico, and to estimate the proportion of infants at risk of ROP who were receiving care in units with ROP programs which complied with Mexican ROP National Guidelines.

## 2. Methods

**Figure 1:**
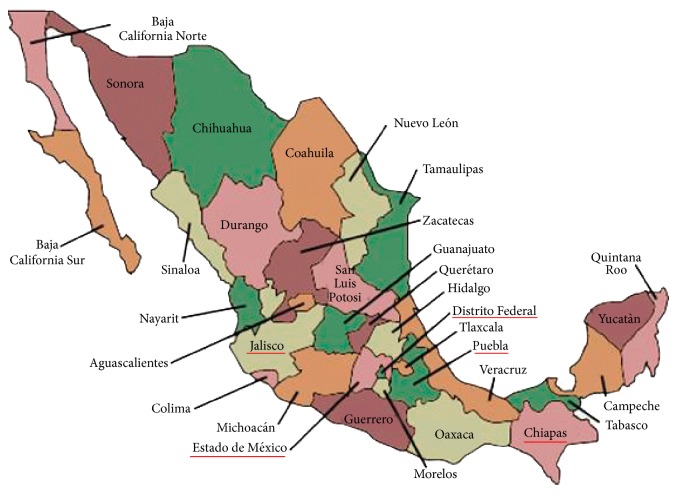
Map of Mexico showing the location of the states included in the study.

The study focussed on NICUs in the public sector and was undertaken in five of the largest states in Mexico, which were purposively selected. The states visited were Mexico State, Puebla, the Federal District where the capital city is located, Jalisco, and Chiapas (Figure 1). These five states account for approximately 37% of all births. In each state a list of NICUs was obtained from the Ministry of Health and these were ranked according to the number of incubators [23], as a proxy for the number of preterm infants admitted. Thirty-five NICUs were selected for the study, with the number selected in each state reflecting the number of known neonatal units in 2006. Two declined to participate and one was a short stay unit. The NICUs were visited between June and August 2011.

In each of the 32 NICUs senior neonatal staff were interviewed after explaining the study and obtaining written informed consent. If the NICU had a program for ROP the following information was obtained: criteria for screening and timing of the first examination; who identified the infants to be screened and who communicated this information to parents; how the infants were screened and where; who performed the screening; whether the infants were treated on the NICU or elsewhere and the method of treatment; which equipment was used for examination and treatment and whether this belonged to the NICU. Information was also sought on who paid for screening and/or treatment. Each NICU was allocated a study number to maintain confidentiality.

### 2.1. Categorization of ROP Programs

Programs were classified as “compliant” if they adhered to the Mexican National ROP Guidelines; that is, a trained ophthalmologist visited the NICU on a regular weekly basis in salaried time to screen infants who fulfilled the eligibility criteria by indirect ophthalmoscopy or if they visited smaller NICUs at the request of the neonatologist when there was an infant to be examined. Either compliant programs provided laser treatment in the NICU or infants were transferred to the eye department. Programs were classified as “noncompliant” if (a) screening did not adhere to the eligibility criteria, (b) it was not undertaken regularly, (c) it was performed by a neonatologist, (d) it was performed by ophthalmologists on a voluntary basis (as this depends on individual's motivation and so is not sustainable), (e) direct ophthalmoscopy was used, or (f) infants were transferred to an eye department for examination. Programs were also classified as noncompliant if primary treatment was with intravitreal Avastin or if parents had to pay for screening and/or treatment. Units were classified as having no program if no screening was undertaken and no infants were treated for ROP.

Data on the number of live births for 2010 were obtained from the Ministry of Health for populations not receiving social security [20]. Numbers of preterm infants at risk were obtained from the Ministry of Health Dynamic Database [20]. Data on neonatal service delivery for 2006, the most recent year, were also obtained from the Ministry of Health to provide information on the NICU's location [23]. All data were collected by one researcher (L. Consuelo Zepeda-Romero).

The study was approved by the ethics committee of the London School of Hygiene & Tropical Medicine and by the Ministry of Maternal and Child Health in Mexico.

## 3. Results

### 3.1. Types and Coverage of ROP Programs

Only 10 units (31.2%) had a fully compliant program, that is, for screening and treatment, but 5 transported infants to the eye clinic for treatment. General hospitals were more likely to have compliant programs (38.9%) than children's or maternity hospitals (33.3% and 12.5%, resp.) (Table 1). A third (34.4%) of study NICUs did not have a program for ROP and a further 34.4% of NICUs had noncompliant programs (Table 1). As the NICUs without ROP programs tended to be smaller, the proportion of the 372 cots without a program was 25.8% with 41.4% being in NICUs with noncompliant programs. The wealthier states (Federal District, Mexico State, and Jalisco) had a higher proportion of compliant ROP programs, and Chiapas and Puebla, the poorer states, had the lowest coverage (Table 2). In Chiapas only one of the six units had a program, which was classified as noncompliant as parents paid for screening and Avastin treatment. In several other NICUs in Chiapas parents were informed that their child was at risk of blindness from ROP and they were advised to take their child to an ophthalmologist.

### 3.2. Details of Procedures for Screening

Twenty-one of the 32 (65.5%) NICUs had some mechanism for screening infants (Table 3). In seven of the general hospitals ophthalmologists from the eye department screened infants in the NICU in salaried time. In four hospitals screening was by ophthalmologists who did this on a voluntary basis, two being maternity hospitals. In one maternity hospital a neonatologist screened infants by indirect ophthalmoscopy and in two NICUs the RetCam imaging system was used to document findings. Infants transferred to the eye department for screening were often not stable enough for transfer until they were over 6 weeks of age. Most of the hospitals provided indirect ophthalmoscopes while the unsalaried ophthalmologists provided their own equipment. In two units where screening was not performed, visually evoked potentials were used to detect ROP at discharge.

#### 3.2.1. Screening Criteria

Amongst the 21 NICUs with an ROP program, 14 (66.6%) followed the Mexican Guidelines but 7 used much narrower criteria; that is, BW ≤1,200 g or GA <32. Two had adjusted the criteria on the basis of their own data but the other five followed criteria from other countries.

### 3.3. Details of Procedures for Treatment

Laser treatment was the most frequent form of treatment (in all 10 compliant programs). Five of the general hospitals owned a laser, and the other ophthalmologists treating with laser either rented the equipment or the infant was transferred and treated in the eye department. Avastin was the treatment of choice in seven units. In six of these units the ophthalmologists were either unsalaried (3 units) or were private practitioners (3 units) (Table 3). In some units Avastin was reportedly used because of lack of anaesthetists.

#### 3.3.1. Who Paid for Screening and Treatment?

In four of the 11 units with a noncompliant program, parents paid for different aspects of care. In three units parents paid private ophthalmologists for screening and treatment with Avastin. In another unit parents paid for transport to the eye clinic for screening and laser treatment, which cost 400 US$. In the 17 other units (i.e., all 10 compliant programs and 7 noncompliant programs) the health provider paid for screening and treatment.

#### 3.3.2. Characteristics of Study Neonatal Intensive Care Units

Twenty-nine of the 32 NICUs visited were in the Secretaria de Salud (SSA) health system, with a total of 345 spaces for intensive neonatal care. This represents approximately a third of the provision of preterm care in Mexico's SSA health system. Two were in the state government health system and one was in the Institute Mexican Social Security (IMSS) health system. Eighteen of the study NICUs were in general hospitals, 8 were in maternity hospitals, and 6 were in children's hospitals. Fifteen of the 24 general or maternity hospitals admitted high risk pregnancies and 11 were referral centres (Table 4).

### 3.4. Other Findings

In several of the general hospitals with eye departments the ophthalmologists reported that infants with Stage 5 ROP were seen on a regular basis, usually from outside the state.

## 4. Discussion

The findings of this study show that much needs to be done to improve the coverage and quality of programs for the detection and treatment of ROP in Mexico, even in the capital city where five of the nine NICUs visited did not have a program. Greater awareness is needed amongst policy makers and planners, so that they are made aware of PAHO priorities for the region and the National ROP Guidelines and that eye examination of preterm infants is a legal requirement. At the time of the study ROP was not included in the National Prevention of Blindness plans for Mexico, despite treatment being covered by national insurance schemes, and this needs to be addressed as a matter of urgency. Greater awareness is also needed amongst the professional organizations of paediatricians, neonatologists, and ophthalmologists so that ROP is included in all residency programs.

Parents need to be better informed about ROP so they know that eye examination of all preterm infants is not only necessary but is a legal requirement of providers (since January 2013) so that they demand services [19]. However, mothers of preterm infants in Mexico are often very young, unmarried, poorly educated, and unemployed [22]. Any educational materials will need to be clear and easy to understand by those with a poor educational background. Better communication will be needed between ophthalmologists and the neonatal team so that mothers can be informed of the need for screening while in the unit and that further examination may be required after discharge.

A shortage of ophthalmologists who are willing to screen is a major constraint in many settings, which has been addressed in other countries, such as Brazil [7], Argentina [8], Chile [9], and China, by policies which allow ophthalmologists to screen in salaried time. However, the complexity of the health system in Mexico makes implementing policies challenging. Many of the ophthalmologists did not visit the unit to screen infants on a regular basis (i.e., on the same day of the week) which makes follow up of discharged infants impossible. In five units ophthalmologists were not using the Mexican criteria for screening [18] but were using the narrower criteria adopted by more advanced economies where the population of infants at risk is very different. Both of these factors make it likely that infants who develop ROP needing treatment are not being screened at all or are not being examined after discharge.

Different approaches to screening are being explored and used, including retinal imaging by neonatologists, nurses, or trained nonphysicians [24], with interpretation of the images at the cot side or through telemedicine [25]. It is likely that new, less expensive cameras suitable for screening preterm infants will soon become available, which offers a paradigm shift in screening, whereby members of the neonatal team could be trained to obtain and interpret retinal images at the cot side, only calling an ophthalmologist to examine infants detected with ROP where treatment may be needed. This approach has the potential to greatly increase the coverage of screening for ROP.

Several units lacked access to a laser, but this problem can be addressed by sharing a laser, as occurs in some cities in other countries. In Mexico this may be challenging, given the different health systems and range of providers. Despite laser treatment being covered by health insurance in Mexico, many providers are not availing themselves of this opportunity as reimbursement processes can be slow and cumbersome, and the fee may go to the hospital rather than the individual providing the treatment. Under these circumstances families are bearing the costs unnecessarily [21, 26]. Many of the families of infants cared for in government facilities are poor, and the out of pocket expenditure for laser treatment is likely to have adverse consequences, but this was not explored in this study. Advocacy will be needed so that health providers cover all costs of the program and to ensure that ophthalmologists are adequately reimbursed for their time. In several units infants were transported to eye clinics for treatment, which is not desirable as it increases stress for the infants, takes staff away from the neonatal unit, and may lead to delay in treatment.

It is also of concern that almost a quarter of the ophthalmologists used Avastin as a first line therapy when so little is known about the long term ocular and systemic complications [27], despite the Mexican Guidelines stating that antiangiogenic agents should only be used when laser treatment has failed [28]. Lack of anaesthetists was the reason given by some, but laser treatment can be delivered safely under topical anaesthesia with sedation in the presence of a neonatologist [29]. Many of the ophthalmologists using Avastin were private or “volunteer” ophthalmologists, where the ease and speed of treatment with Avastin compared with laser may be a factor, but this was not explored. There needs to be greater awareness of the potential complications of Avastin among neonatologists and ophthalmologists until further trials indicate that lower doses or different preparations are not only effective but safe.

A limitation of this study is that it only included NICUs in state capital cities. However, as the majority of ophthalmologists in Mexico work in the major cities, the provision of ROP programs is likely to be even poorer outside state capitals. The present study provides a baseline which can be used to assess change in the coverage and quality of ROP programs over time in response to change in policies and greater awareness amongst service providers and parents.

## Figures and Tables

**Table 1:** Type of program for ROP in 32 neonatal units in five states in Mexico, including number and proportion of cots.

	Compliant program			Noncompliant program			No program		
	*N*	% of units	% of cots	*N*	% of units	% of cots	*N*	% of units	% of cots
General hospital *N* = 18 (191 cots)	7 (87)	*38.9% *	45.5%	4 (50)	*22.2% *	26.2%	7 (54)	*38.9% *	28.3%
Maternity hospital *N* = 8 (128 cots)	1 (16)	*12.5% *	*12.5% *	5 (82)	*62.5% *	64.1%	2 (30)	*25% *	23.4%
Children's hospital *N* = 6 (53 cots)	2 (19)	*33.3% *	*35.8% *	2 (22)	*33.3% *	*41.5% *	2 (12)	*33.3% *	22.6%
All NICUs *N* = 32 (372 cots)	10 (122)	31.2%	32.8%	11 (154)	34.4%	41.4%	11 (96)	34.4%	25.8%

**Table 2:** Type of program in neonatal units in five states in Mexico, ranked by wealth status.

States ranked by wealth	Compliant program		Noncompliant program		No program		Total	
	*N*	%	*N*	%	*N*	%	*N*	%
Federal District	3	33%	1	11%	5	56%	9	100%
Mexico State	3	38%	4	50%	1	13%	8	100%
Jalisco	3	60%	2	40%	0	0%	5	100%
Puebla	1	25%	3	75%	0	0%	4	100%
Chiapas	0	0%	1	17%	5	83%	6	100%
**Total**	**10**	**31.2%**	**11**	**34.4%**	**11**	**34.4%**	**32**	**100%**

**Table 3:** Approach to screening and treatment in 32 neonatal units in five states in Mexico.

	*Detection of ROP requiring treatment *						Total
	Salaried ophthalmologist visits NICU regularly	Private ophthalmologist visits NICU when requested	Unsalaried ophthalmologist visits NICU regularly	Examination in eye unit or elsewhere	Examined by neonatologist	No eye examination	
*Method and location of treatment *							
Laser photocoagulation in the NICU	5^*∗∗∗*^	0^*∗∗∗*^	1^*∗∗*^	0^*∗∗*^	0^*∗∗*^	*∗*	**6**
	General hospital 3; maternity hospital 1; children's hospital 1		General hospital 0; maternity hospital 1; children's hospital 0				
Laser treatment in the eye unit or elsewhere	4^*∗∗∗*^	1^*∗∗∗*^	0^*∗∗*^	2^*∗∗*^	1^*∗∗*^	*∗*	**8**
	General hospital 3; maternity hospital 0; children's hospital 1	General hospital 1; maternity hospital 0; children's hospital 0		General hospital 1; maternity hospital 0; children's hospital 1	General hospital 0; maternity hospital 1; children's hospital 0		
Avastin as first line treatment	1^*∗∗*^	3^*∗∗*^	3^*∗∗*^	0^*∗∗*^	0^*∗∗*^	*∗*	**7**
	General hospital 1; maternity hospital 0; children's hospital 0	General hospital 2; maternity hospital 0; children's hospital 1	General hospital 0; maternity hospital 1; children's hospital 2	—	—		
No treatment	*∗*	*∗*	*∗*	*∗*	*∗*	11 NICUs^*∗*^ General hospital 7; maternity hospital 2; children's hospital 2	**11**
Total	**10**	**4**	**4**	**2**	**1**	**11**	**32**

^*∗∗∗*^Compliant ROP program.

^*∗∗*^Noncompliant ROP program.

^*∗*^No ROP program.

**Table 4:** Number of neonatal units included in the study by hospital type and the proportion of all cots in each state included in the study.

Study states	General hospital	Maternity hospital	Children's hospital	Total visited	% of cots in state in 2010
Federal District	3	3	3	**9**	42.7%
Mexico State	4	3	1	**8**	56.2%
Jalisco	4	1	0	**5**	46.3%
Puebla	2	1	1	**4**	42.3%
Chiapas	5	0	1	**6**	38.4%
**Total**	**18**	**8**	**6**	**32**	**45.2%**

## Abbreviations

SSA: Secretaria de Salud Mexico
IMSS: Instituto Mexicano del Seguro Social
BW: Birth weight
GA: Gestational age.
